# Frontal white matter lesions in Alzheimer’s disease are associated with both small vessel disease and AD-associated cortical pathology

**DOI:** 10.1007/s00401-021-02376-2

**Published:** 2021-10-04

**Authors:** Kirsty E. McAleese, Mohi Miah, Sophie Graham, Georgina M. Hadfield, Lauren Walker, Mary Johnson, Sean J. Colloby, Alan J. Thomas, Charles DeCarli, David Koss, Johannes Attems

**Affiliations:** 1 Translation and Clinical Research Institute, Newcastle University, Newcastle upon Tyne, NE4 5PL UK; 2 Department of Neurology, University of California, Davis, CA USA

**Keywords:** White matter lesion, White matter hyperintensity, Alzheimer’s disease, Small vessel disease, Amyloid-beta, Hyperphosphorylated tau

## Abstract

Cerebral white matter lesions (WML) encompass axonal loss and demyelination and are assumed to be associated with small vessel disease (SVD)-related ischaemia. However, our previous study in the parietal lobe white matter revealed that WML in Alzheimer’s disease (AD) are linked with degenerative axonal loss secondary to the deposition of cortical AD pathology. Furthermore, neuroimaging data suggest that pathomechanisms for the development of WML differ between anterior and posterior lobes with AD-associated degenerative mechanism driving posterior white matter disruption, and both AD-associated degenerative and vascular mechanisms contributed to anterior matter disruption. In this pilot study, we used human *post-*mortem brain tissue to investigate the composition and aetiology of frontal WML from AD and non-demented controls to determine if frontal WML are SVD-associated and to reveal any regional differences in the pathogenesis of WML. Frontal WML tissue sections from 40 human *post-mortem* brains (AD, *n = *19; controls, *n = *21) were quantitatively assessed for demyelination, axonal loss, cortical hyperphosphorylated tau (HPτ) and amyloid-beta (Aβ) burden, and arteriolosclerosis as a measure of SVD. Biochemical assessment included Wallerian degeneration-associated protease calpain and the myelin-associated glycoprotein to proteolipid protein ratio as a measure of *ante-mortem* ischaemia. Arteriolosclerosis severity was found to be associated with and a significant predictor of frontal WML severity in both AD and non-demented controls. Interesting, frontal axonal loss was also associated with HPτ and calpain levels were associated with increasing Aβ burden in the AD group, suggestive of an additional degenerative influence. To conclude, this pilot data suggest that frontal WML in AD may result from both increased arteriolosclerosis and AD-associated degenerative changes. These preliminary findings in combination with previously published data tentatively indicate regional differences in the aetiology of WML in AD, which should be considered in the clinical diagnosis of dementia subtypes: posterior WML maybe associated with degenerative mechanisms secondary to AD pathology, while anterior WML could be associated with both SVD-associated and degenerative mechanisms.

**Supplementary Information:**

The online version contains supplementary material available at 10.1007/s00401-021-02376-2.

## Introduction

Cerebral white matter lesions (WML) histologically encompass rarefaction of the cerebral white matter, primarily presenting as demyelination with/without axonal loss with associated oedema and reactive gliosis [17]. During life, white matter changes are detected radiologically as white matter hyperintensities (WMH) on T2-weighted magnetic resonance imaging (MRI) [19] or as altered diffusivity on diffusion tensor imaging (DTI) [6], although it important to note that the presence of a WMH cannot inform us of the underlying causative pathology. WML/WMH are highly associated with age [46], and are commonly seen in individuals with and without dementia [47] presumed to reflect chronic ischaemia-associated demyelination and axonal loss due to arteriopathy of the perforating white matter arteries and arterioles, i.e., type 1 arteriolosclerosis cerebral small vessel disease (SVD) [41, 42]. SVD itself is heterogeneous incorporating atherosclerosis, lipohyalinosis, arteriolosclerosis, blood-brain barrier (BBB) breakdown, fibroid necrosis, and calcification of the perforating arteries and/or arterioles. The presence of WMH is often interpreted as a surrogate marker for SVD [13, 15]. Furthermore, cerebral amyloid angiopathy (CAA), characterized by the accumulation of amyloid-beta (Aβ) pathology in leptomeningeal, cortical, and capillary vessel walls (Type 2 cerebral SVD [42]), has been shown to be associated with white matter disruption and increased WMH volume [1, 31] as well as increased demyelination of associated white matter [53].

WMH are more frequent and severe in Alzheimer’s disease (AD) compared to other neurodegenerative diseases and normal ageing [4, 29, 58] with increased WMH volume contributing to the progression and severity of the clinical syndrome [9, 38]. The pathogenesis of WML/WMH in AD is complex and poorly understood, incorporating multiple aspects of the neurovascular unit, neurodegeneration, neuroinflammation, and vascular risk factors [39, 45] as well as vascular changes. Pioneering neuropathological studies in AD and non-AD tissue first indicated that WM changes in AD may result from both ischaemia, particularly in the frontal region [14], or from secondary axonal degeneration associated with AD pathology in the overlying cortex, particularly the temporal and parietal regions [10, 26]. Following on, numerous neuroimaging and neuropathological studies [1, 21, 25, 29, 30, 48] have suggested that WMH in the medial-temporal lobe and posterior regions of the brain can also occur as a result of degenerative white matter changes attributed to the deposition of cortical AD pathology, i.e., hyperphosphorylated tau (HPτ) and Aβ. We previously performed a comprehensive quantitative neuropathological investigation of the composition and pathogenesis of WML in parietal white matter from individuals clinico-pathological confirmed as AD, as well as age-matched non-demented controls [30]. We revealed that pathological WML in AD are associated with both axonal loss and demyelination, in contrast to non-demented individuals which were primarily associated with demyelination only, and severity of WML are associated with HPτ deposition. Furthermore, we biochemically measured the Wallerian degeneration-specific protease calpain that is thought to be activated by AD pathology-related axonal transport dysfunction and is associated with the retrograde degradation of axonal cytoskeletal proteins [12, 28]. Analysis revealed that calpain was significantly higher in WML tissue of AD cases and was associated with AD pathology burden in the overlying cortex. Overall, this previous study indicated that in the parietal white matter of AD cases, WML can result as a consequence of degenerative axonal loss secondary to AD pathology in addition to SVD-associated white matter changes, in agreement with the neuroimaging studies. The findings of our study were confirmed by subsequent *post-mortem* studies from other groups; Kantarci et al. [21] revealed that Braak NFT staging of HPτ was associated with *ante-mortem* DTI alterations in parietal white matter and medial-temporal lobe connections, and Alosco et al.[1] revealed *ante-mortem* WMH volume increased the odds of having a higher degree of AD-related pathology at autopsy. In addition, *ante-mortem* imaging has indicated that tau PET ([^18^F] AV-1451) was associated with DTI changes in the anterior temporal region and tau pathology independently contributed to WM changes [51].

Efforts are now focused on investigating regional differences in the aetiology of WML. The frontal white matter region has been shown to be particularly sensitive to cardiovascular risk factors (CVRF) with increased frontal WMH volume associated with CVRF of middle age [44]. In addition, due to the topographical rostral progression of HPτ pathology from the entorhinal/limbic regions to the neocortex, the frontal region is affected in the rather late stages of AD progression, and therefore, in early stages of the disease the frontal white matter, theoretically is less likely to develop degenerative axonal loss compared to the posterior white matter. A pivotal DTI study by Lee et al. further investigated the rostral-caudal gradient of WMH within the corpus callosum (CC) [25]; data from this study indicate that both AD-associated degenerative mechanisms and vascular processes contributed to white matter disruption within the anterior CC, in contrast to disruption in the posterior CC that was primarily driven by AD-associated degeneration. The prominent influence of AD-associated degenerative mechanism in the posterior regions is in agreement with our previous findings from the parietal white matter [30].

It is yet to be determined in human tissue if the pathogenesis of frontal WML is primarily associated with vascular processes, i.e., SVD and CAA, AD-associated degenerative axonal loss, or a mixture of both. Therefore, in this pilot study, we aimed to identify differences in the composition and aetiology of WML in the frontal lobe of AD and non-demented controls using pathological and biochemical methods. Based on our previous neuropathological study and indications from previous imaging studies, we hypothesise that both pathological measures of SVD and HPτ pathology and biochemical measures of ischaemia and axonal degeneration are associated with and predictors of frontal WML severity and pathological changes, i.e., axonal loss and demyelination.

## Methods

**Fig. 1:**
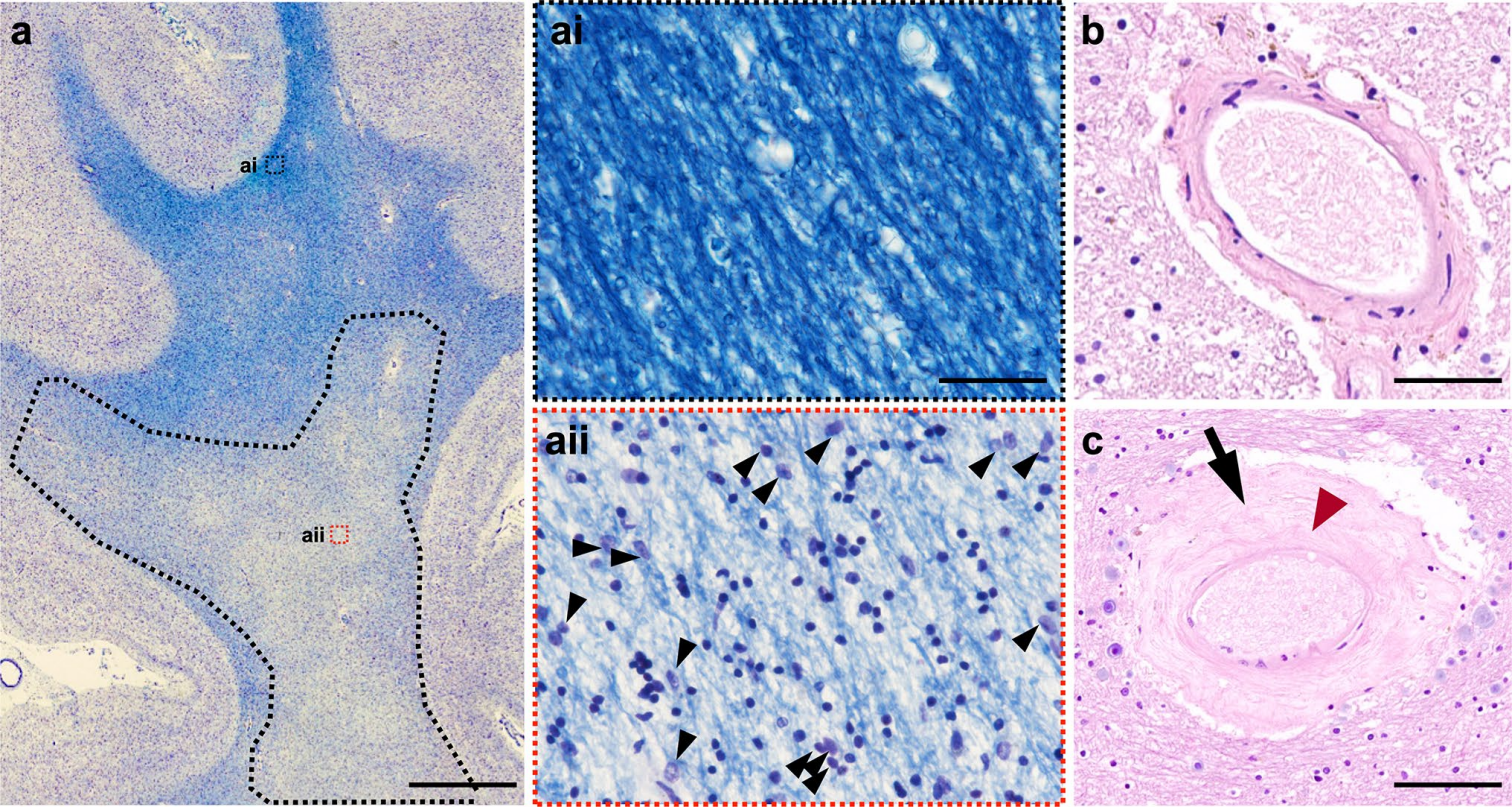
**a** LFB and hematoxylin-stained section of frontal lobe tissue illustrating a white matter lesion in the deep white matter (black dashed line; note pallor). (ai) Magnified photoimage of normal appearing white matter; (aii) magnified image of white matter lesion area showing rarefaction of tissue and associated gliosis (black arrow heads); **b** normal white matter arteriole; **c** white matter arteriole with severe arteriolosclerosis exhibiting vessel wall hyalinosis (black arrow) and complete loss of smooth muscle cells (red arrowhead). LFB, luxol fast blue; SVD, small vessel disease; *Scale bar,* 0.5 cm valid for image **a**; 20 μm valid for ai and aii; 50 μm valid for image **b**; 100 μm valid for image **c**

Our study cohort consisted of 40 consecutive donated human *post-mortem* brains (mean age 84.7 ± 4.9 years; male: 16, female: 24) which were recruited through tertiary research centers or memory clinics. This cohort was based on a subset of cases from the same cohort previously used to investigate parietal WML [30] and cases were subdivided based on the clinico-pathological diagnosis. The known causes of death are listed in Supplementary table 1. During life, all dementia subjects and 18/21 (85.7%) of the non-demented control subjects underwent prospective clinical assessments by board-certified Old Age Psychiatrists or Neurologists. All cases had a clinical review of previous psychiatric assessment, as well as a collateral interview to determine cognitive status and the reading of medical records if clinical information for non-demented controls was required after death at Newcastle Brain Tissue Resource (AJT). Non-demented control subjects were confirmed as having showed no evidence of cognitive impairment and had normal everyday functioning up until death. AD cases were confirmed as having a clinical dementia due to AD using the NIA-AA criteria [33, 34]. Brain tissue was obtained at autopsy and stored within the Newcastle Brain Tissue Resource in accordance with Newcastle University Ethics Board (The Joint Ethics Committee of Newcastle and North Tyneside Health Authority, reference: 08/H0906/136). After autopsy, the left hemisphere, brainstem, and cerebellum were dissected in coronal planes approximately 0.7 cm intervals and snap frozen between copper plates at − 120 °C and stored at − 80 °C. The right hemisphere, brainstem, and cerebellum were immersion fixed in 10% buffered aqueous formaldehyde solution for 4 weeks and then subsequently dissected in coronal planes approximately 0.7 cm intervals and paraffin-embedded. All brains underwent neuropathological assessment according to the National Institute on Ageing-Alzheimer’s Association (NIA-AA) criteria [36], inclusive of Thal phases of Aβ deposition [52], Braak staging of neurofibrillary pathology [8], and Consortium to Establish a Registry for Alzheimer’s Disease scoring [35]. Additional neuropathological scoring of Lewy body pathology [7, 32], vascular pathology contribution to cognitive impairment (vascular impairment neuropathological guidelines (VCING); this was inclusive of semi-quantitate assessment of SVD and WML) [49], TDP-43 inclusions for the assessment of limbic-predominant age-related TDP-43 encephalopathy neuropathological change (i.e., LATE-NC) [20] and CAA [40] was performed. Cases did not contain any large infarcts (> 1 cm) or cerebral hemorrhages. The final clinico-pathological diagnosis was AD in 19 cases and non-demented controls in 21 cases. Of note, SVD and other vascular pathologies and/or lesions maybe present in the AD cases, but it did not fill the neuropathological criteria (severe level) to be deemed a mixed dementia in which both the AD and SVD/vascular pathology could independently lead to clinical dementia. Examples of normal appearing white matter, a WML, and SVD pathology are presented in Fig. 1.

### Histological procedures

Paraffin-embedded serial sections from pre-frontal blocks (Brodmann area 9) were cut at 6 μm thickness and mounted onto superfrost plus charged glass slides (Thermo Shandon, Cheshire, UK). These sections of tissue primarily contained juxtacortical and deep white matter. Any residual periventricular white matter was excluded from analysis. Sections underwent histological staining with Bielschowsky silver stain (cold method) [27] for assessment of axonal density, myelin stain luxol fast blue (LFB) to assess WML area and demyelination and haematoxylin and eosin (H&E) for visualisation of white matter artery/arteriole walls for assessment of SVD. Immunohistochemistry was performed for HPτ (antibody AT8: pSer199/202/thr205; Innogenetics, Belgium: mouse monoclonal; 1:4000) and Aβ peptide (antibody 4G8: Amyloid 17–24; Signet Labs, Dedham, MA, USA: mouse monoclonal; 1:15,000). Prior to immunostaining, antigen retrieval was performed by microwaving slides in 0.01 mL citrate buffer for 10 min for AT8 and immersion in concentrated formic acid for 1 h for 4G8. Immunopositivity was detected using the Menarini X-Cell-Plus HRP Detection Kit (Menarini Diagnostics, Winnersh-Wokingham, UK) with 3,3 diaminobezidine (i.e., DAB) as a chromagen and haematoxylin as a counter stain. All histologically and immunohistochemically stained sections were subsequently dehydrated through a series of alcohols, and cleared and mounted using DPX (CellPath, Powys, UK).

### Image analysis

#### White matter lesion area and demyelination

All image analysis protocols are described in detail in [30]. All image analysis was performed blind to clinical diagnosis. Briefly, all whole LFB sections were scanned using an Epson Perfection V700 scanner and monochrome images (8-bit grey scale) uploaded into Image-Pro Plus software program (Media Cybernetics Inc, USA; version 6.3). White matter area was manually delineated from the cortical ribbon using the freehand selection tool and total white matter area recorded. The WML area was identified by eye based on a defined reduction in grey colour that is clearly differentiated from the surrounding normal appearing white matter (NAWM), and was confirmed by microscopic examination by the identification of white matter pallor, vacuolation, loosening of tissue, and gliosis [50]. To quantify the WML area from the normal appearing white matter (NAWM), the 8-bit grey scale threshold was manually adjusted to select only the WML area. The percentage area of WML per total white matter area was calculated and expressed as percentage WML area (WMLA) to obtain a measure of overall WML severity. The integrated optical density (IOD) of the LFB stain was determined as a measure of demyelination. The IOD represents the amount of light transmitted through a sample, and therefore, a higher value signifies a greater amount of light transmission, i.e., a lighter stain and a reduction of myelin. Three images from the WML and three images from the NAWM were randomly captured at 200 × magnification using a Nikon 90i microscope and uploaded on Image-Pro Plus. The mean IOD per pixel was recorded for each image and a subsequent mean IOD per pixel for WML and NAWM per case were calculated.

#### Axonal density

Once a WML was identified on an LFB section, the WML area was roughly delineated by hand using a permanent marker and was used to identify the same corresponding WML area in the adjacent Bielschowsky stained section. Within the WML area and NAWM on the Bielschowsky section, five randomly selected areas had 3 × 3 single images at 200 × magnification which were captured and using NIS elements version 3.0 (Nikon, Surrey, UK). Individual and standardized Red Green Blue (RGB) thresholds for Bielschowsky’s staining were applied until all visible axons were included with necessary manual setting of regions of interest to exclude vessels and perivascular spaces and artifacts. The area covered by Bielschowsky’s stain was measured and its percentage of the total measured area is calculated and expressed as Bielschowsky’s area (BiA), and mean regional values were calculated for the WML area and NAWM per case.

#### Quantification of HPτ and Aβ pathology

**Fig. 2:**
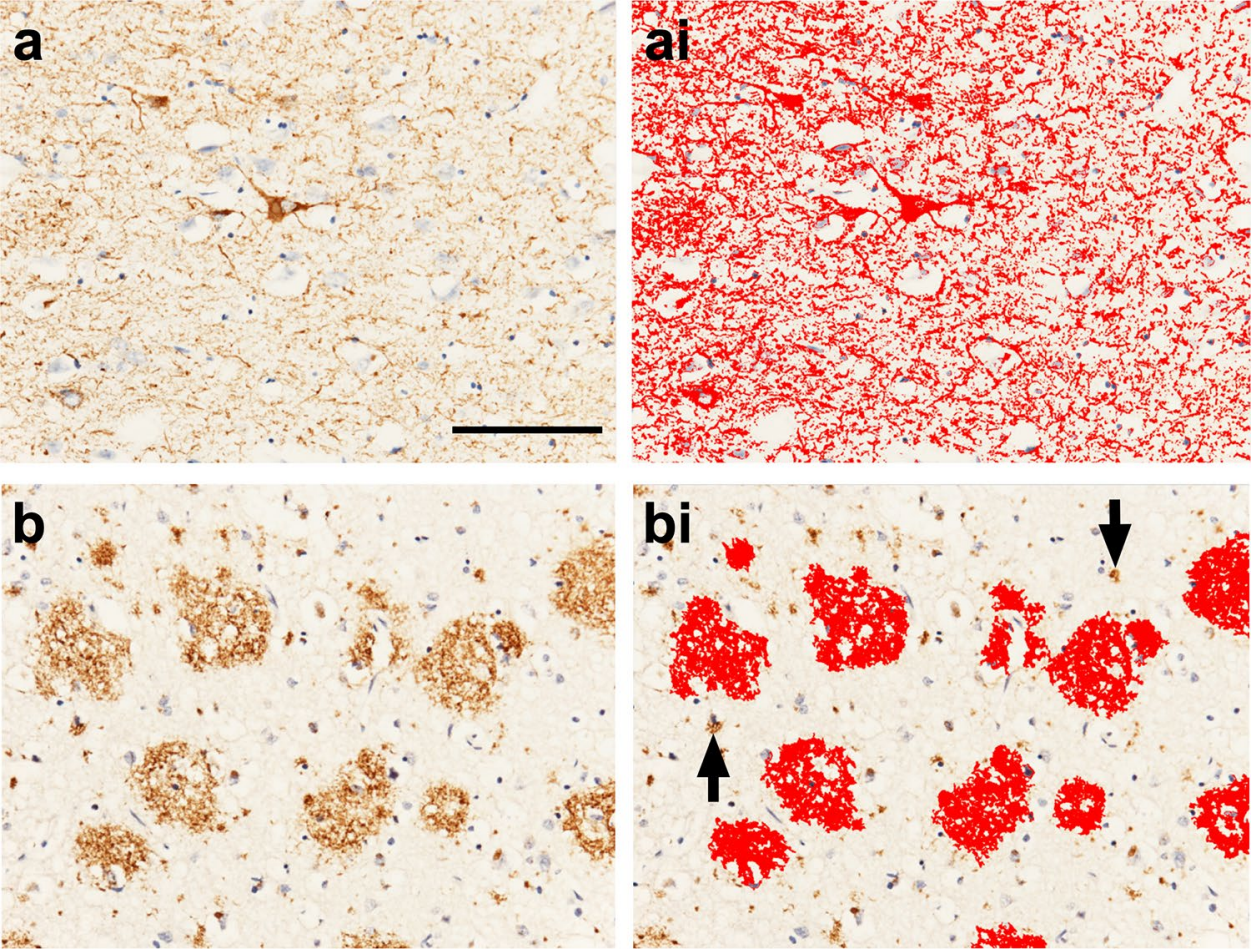
**a** 3 × 3 large image acquisition of frontal cortical AT8-IR (HPτ pathology). (ai) Image A with applied bespoke AT8-IR threshold (red outline). **b** 3 × 3 large image acquisition of frontal cortical 4G8-IR (Aβ pathology). (bi) Image b with applied bespoke 4G8-IR threshold (red outline) inclusive of size restriction to eliminate the measurement of physiological cellular APP (black arrows). Mean area covered by IR was stated as a percentage of the total image area and the respective values are expressed as AT8-IR or 4G8-IR. IR, immunoreactivity. *Scale bar,* 100 μm valid for all images

3 × 3 single images were captured at 200 × magnification in three frontal cortex sample areas inclusive of the sulci, mid-cortical ribbon, and gyri. Standardized Red Blue Green (RGB) thresholds were applied separately for AT8 and 4G8 RGB intensity values for binary layer pixels were set as follows: AT8: R25-170, G27-156, B11-126; 4G8: R50-180, G20-168, and B8-139. In addition, we set a size restriction threshold for the assessment of 4G8, which excluded the measurement of immunoreactive signals with an area below 100μm^2^; this was necessary to ensure that physiological APP that is stained with 4G8 antibody was not included in the measurement. The percentage areas covered by AT8 and 4G8 immunoreactivity were measured and the mean values for the four sample areas were calculated and are expressed as AT8-IR and 4G8-IR, respectively (Fig. 2).

#### Quantification of SVD

Sclerotic index (SI) is a quantitative measure of arteriolosclerosis in the context of SVD. In this study, SI assessment was restricted to white matter arteries and arterioles only. SI is calculated using the formula SI = 1—(internal diameter/external diameter); the SI of normal arteries and arterioles ranges from 0.2 to 0.3, while an SI of 0.3–0.5 indicates mild-to-moderate SVD and SI values > 0.5 are seen in severe SVD [23]. Using H&E sections, a maximum of eight randomly selected cerebral white matter arteries and/or arterioles were identified, and a single image captured at 200 × magnification. Images were uploaded to the software program VasCalc [56] and SI per vessel calculated followed by the calculation of a mean SI value per case.

### Biochemistry

All protocols are described in detail in [30]. Briefly, 10 μm frozen sections inclusive of white matter from Brodmann area 9 were cut, fixed and histochemically stained with LFB to visualise any WML present; this was then used to guide frozen tissue extraction of approximately 250 mg of WML tissue and approximately 250 mg of NAWM tissue from the frozen coronal slab incorporating Brodmann area 9. Lysis buffer contained 0.2 M tetraethyl ammonium bicarbonate, pH 7.2 (i.e., TEAB; Sigma, UK), protease inhibitor tablets (1 tablet per 10 ml; Complete, Roche, Burgess Hill, UK) and PhosSTOP, phosphatase inhibitor tablets (1 tablet per 10 ml, Roche) were added to tissue samples at a 1 mg/1 ml ratio and samples were homogenised using an Ultra-turrax T10 homogeniser (S10N-5G 5 mm-diameter probe; 30,000 rpm), aliquoted at 500 μl with 5 μl of 10% SDS. Total protein concentration was determined by Bradford assay and final protein concentration for all samples was adjusted to 3ug/ul. Using commercially available enzyme-linked immunosorbent sandwich assays (ELISA) kits, three proteins were measured in both WML and NAWM tissue samples, including Wallerian degeneration markers calpain2 (CAPN2; large catalytic subunit (80 kDa) found in myelinated axons), and ischaemia markers myelin-associated glycoprotein (MAG; myelin sheath protein, expressed only in the myelin loops and highly vulnerable under ischaemic conditions) and proteolipid protein (PLP; myelin sheath protein that is abundant throughout the sheath and stable under ischaemic conditions) [5] to calculate the MAG:PLP as an indication of *pre-mortem* WM ischaemia [5]. Details regarding ELISA kits are presented in Table 1. ELISA protocols were followed according to manufacturer’s instructions as previously described [30].

**Table 1:** ELISA kits used for detection of calpain2, MAG and PLP proteins

Protein	ELISA kit		Sample dilution	Standard curve (ng/ml)	OD wavelength
	Cat #	Description			
CAPN2	CSB-E17822h	Human CAPN2 ELISA kit, Cusabio	Neat samples	2.5–0.039	450 and 540 nm
MAG	CSB-E17901h	Human MAG ELISA Kit, Cusabio	Neat samples	20–0.1	450 and 540 nm
PLP	MBS266920	Human PLP ELISA Kit, MyBioSource	Neat samples	10–0.156	450 nm

*CAPN2*, calpain2; *MAG*, myelin-associated glycoprotein; *PLP*, proteolipid protein

### Statistics

The Statistical Package for Social Sciences software (SPSS ver. 27) was used for statistical evaluation. Variables were tested for normality using the Shapiro–Wilk test and visual inspection of variable histograms. Differences in related variables (WML vs. NAWM) were assessed using a parametric paired t test or non-parametric Wilcoxon test. Group effects were assessed using either parametric (independent samples *t* test) or non-parametric (Mann–Whitney *U*) procedures. Where appropriate, Pearson’s (*r*) or Spearman’s (*ρ*) correlation coefficients, including pairwise deletion, were used to assess associations between variables. Relationships between categorical variables were explored using a *χ *^2^. Stepwise linear regression analyses, inclusive of pairwise deletion and collinearity diagnostics (inclusive of variance inflation factor (VIF), were also conducted to investigate if independent pathological variables of SVD, AD pathology, or CAA are predictors of dependent variables of WML severity, axonal loss and demyelination, ischaemia, or Wallerian degeneration. Statistical threshold for significance was set to *p < *0.05.

## Results

### Inter- and intragroup comparisons

No significant differences were observed in age (*p = *0.470) or *post-mortem* delay (*p = *0.789) between AD and control groups. Age was not associated with WMLA, BiA (axonal density), LFB-IOD (i.e., demyelination), SI (i.e., arteriolosclerosis severity), CAA, AT8-IR (HPτ), or 4G8-IR (i.e., Aβ) (all *p > *0.072). All 40 cases underwent NAWM assessment and 29 cases (16 AD; 13 controls) exhibited at least one WML. AD cases had significantly higher levels of both semi-quantitative and quantified levels of AD neuropathology (all *p < *0.0001), CAA severity (*p = *0.001) compared to non-demented controls. No differences in SI values, WMLA, or NAWM/WML measures of BiA or LFB-IOD were revealed between AD and controls (*p > *0.329) (all data are presented in Table 2).

**Table 2:** Demographic and neuropathological characteristics of study cohort

	AD	Control	Statistic_*(df)*_, *p* value
Cohort number	19	21	
Mean age, years (± SD)	84.68 (4.87)	86.19 (7.70)	*t*_(38)_ = 0.731, *p = *0.470
Gender and mean age, years (± SD)	Male, *n = *6; 85.33 (5.35) Female, *n = *13; 84.39 (4.82)	Male, *n = *10; 83.15 (5.94) Female, *n = *11; 89.09 (8.92)	M:F; Fischer’s_(1)_, *p = *0.239
Mean PMD, hours (± SD)	54.07 (24.19)	51.54 (25.22)	*t*_(26)_ = − 0.270, *p = *0.789
Thal Aβ phase [52]	Phase 4, *n = *1 Phase 5, *n = *18	Phase 0, *n = *6 Phase 1, *n = *7 Phase 2, *n = *4 Phase 3, *n = *1 Phase 4, *n = *3	U_(38)_ = 16.5, *p = *0.0001
Braak NFT stage [8]	NFT stage V–VI, *n = *19	NFT stage 0, *n = *1 NFT stage I-II, *n = *8 NFT stage III-IV, *n = *12	U_(38)_ = 0.500, *p = *0.0001
CERAD [35]	C, *n = *19	Negative, *n = *16 A, *n = *3 B, *n = *2	–
NIA-AA [36]	High, *n = *19	No, *n = *6 Low, *n = *13 Intermediate, *n = *2	–
McKeith criteria [32]	No LBD, *n = *15 Brainstem, *n = *3 Amygdala predominant, *n = *1	No LBD, *n = *18 Brainstem, *n = *3	–
VCING criteria [49]	Low, *n = *19	Low, *n = *21	–
LATE-NC [20]	Present, *n = *13 Absent, *n = *6	Present, *n = *5 Absent, *n = *16	-
CAA score [40]	Stage 0, *n = *3 Stage 1, *n = *4 Stage 2, *n = *5 Stage 3, *n = *7 CapCAA absent, *n = *14 CapCAA present, *n = *5	Stage 0, *n = *14 Stage 1, *n = *1 Stage 2, *n = *5 Stage 3, *n = *1 CapCAA absent, *n = *21	–
WMLA % (± SD)	27.92 (21.52)	27.24 (17.37)	*t*_(38)_ = − 0.111, *p = *0.912
SI (± SD)	0.3 (0.076)	0.28 (0.067)	*t*_(38)_ = 0.990, *p = *0.329
AT8-IR (± SD)	11.50 (17.75)	0.08 (0.154)	U_(38)_ = 8.5, *p = *0.0001
4G8-IR (± SD)	16.10 (10.51)	3.00 (5.98)	U_(38)_ = 29.00, *p = *0.0001
NAWM BiA (± SD)	87.2 (6.38)	88.7 (4.96)	t_(38)_ = 0.794, *p = *0.433
WML-BiA (± SD)	64.37 (12.86)	63.04 (10.29)	*t*_(11)_ = 0.205, *p = *0.839
NAWM LFB-IOD (± SD)	82.9E7 (8.81E7)	81.7E7 (5.87E7)	*t*_(38)_ = 0.496, *p = *0.623
WML LFB-IOD (± SD)	77.4E7 (6.19E7)	79.15E7 (5.73E7)	*t*_(11)_ = 0.891, *p = *0.378

*AD*, Alzheimer’s disease; *df*, degrees of freedom; *t*, Independent samples test; *F*, female; *M*, Male; *U*, Mann–Whitney *U* test; PMD, *post-mortem* delay; *Aβ*, amyloid-beta; *NFT*, neurofibrillary tangle; CERAD, Consortium to Establish a Registry for Alzheimer's Disease; *NIA-AA*, National Institute on Ageing—Alzheimer’s Association criteria for AD neuropathologic change; *LB*, Lewy body; *VCING*, vascular cognitive impairment neuropathological guidelines; *LATE-NC*, limbic-predominant age-related TDP-43 encephalopathy neuropathological change; *CAA*, cerebral amyloid angiopathy; *CapCAA*, capillary CAA; *WMLA %*, white matter lesion percentage area; *SI*, Sclerotic Index; *IR*, immunoreactivity

Intragroup comparisons of whole cohort and separate AD and control NAWM and WML tissue measures indicated that WML tissue had significantly lower BiA (all *p < *0.0001) and significantly higher LFB-IOD values (all *p < *0.05) compared to NAWM, indicating higher levels of axonal loss and demyelination in the WML area as expected.

### Cardiovascular risk factors

Recorded CVRF including presence of hypertension, diabetes, cardiovascular disease and smoking status, as well as final MMSE scores are presented in Supplementary table 1. Employing *χ *^2^ test adjusted for age, the presence of hypertension was not significantly associated with semi-quantitatively assessed SVD score in either AD (*p = *0.333) or the control group (*p = *0.259) nor with semi-quantitatively assessed WML (AD, *p = *0.634; controls *p = *0.625).

### Associations between WML severity and WML measures of axonal loss and demyelination

**Fig. 3:**
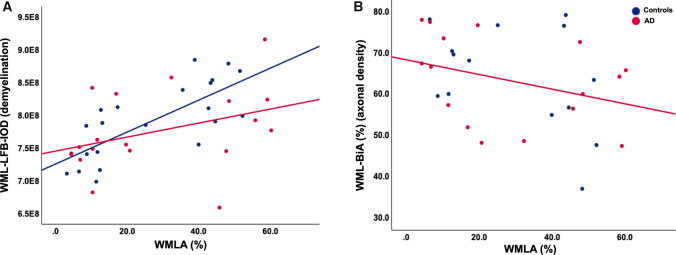
Scatter graphs **a** A correlation between WMLA (i.e., white matter lesions severity) and WML-LFB-IOD (i.e., demyelination) was seen in both AD and control cases. **b** A negative correlation between WMLA and WML-BiA (axonal density) in AD cases only. p values and associated correlation coefficients are shown in main text. WML, white matter lesion; WMLA, white matter lesion area; LFB-IOD, luxol fast blue integrated optical density (demyelination); BiA, Bielschowsky’s area (axonal density decrease)

Using Pearson’s (*r*) or Spearman’s (*ρ*) correlation coefficients, we examined the relationship between WML measures of axonal density (BiA) and myelin loss (LFB-IOD) adjusting for age; no significant association was revealed between BiA and LFB-IOD in either the AD (*r = *− 0.375, *p = *0.084) or the control group (*r = *− 0.114, *p = *0.356). We then examined the relationship between WMLA and WML measures of BiA and LFB-IOD; regarding the whole cohort, WMLA negatively correlated with BiA (*r = *− 0.386, *p = *0.019) and LFB-IOD (*r = *0.541, *p = *0.001), indicating that increase WML severity was associated with increasing axonal loss and demyelination. However, when dichotomized based on diagnosis, WMLA in the control cases was only associated with LFB-IOD (*r = *0.698, *p = *0.004; Fig. 3a) and not BiA (*p = *0.079; Fig. 3b), whereas WMLA in AD cases was associated with both LFB-IOD (*r = *0.457, *p = *0.033; Fig. 3a) and BiA (*r = *− 0.479, *p = *0.031; Fig. 3d). This indicates that WMLA seen in the control cases are primarily associated with demyelination and WMLA seen in AD cases are associated with both axonal and independent/axonal loss-associated demyelination.

### Influence of SVD, CAA, and cortical neurodegenerative pathology on WML severity axonal loss and demyelination

We investigated whether cerebrovascular pathologies, i.e., SVD (SI) and CAA, and cortical AD pathologies, i.e., HPτ (AT8-IR) or Aβ (4G8-IR) were associated with white matter pathological changes, i.e., WML severity (WMLA), axonal density (BiA), and myelin loss (LFB-IOD). In the AD group, SI was significantly associated with WMLA (*r = *0.571, *p = *0.002) and LFB-IOD (− 0.597, *p = *0.009) but not BiA (*p = *0.103). In the controls, SI was also associated with WMLA (*r = *0.648, *p = *0.001) and WML LFB-IOD (*r = *0.707, *p = *0.003) and not WML-BiA (*p = *0.206). CAA was not associated with WMLA, WML-BiA, or WML-LFB-IOD in either the AD or control group (*p > *0.408). Regarding cortical AD pathologies, only WML-BiA in the AD group was associated with AT8-IR (*ρ* = 0.432, *p = *0.037). No other association between AT8-IR and 4G8-IR were revealed with any variable in any group (*p > *0.074).

Stepwise linear regression with pairwise deletion and controlled for age was used to investigate if the independent variables SI, CAA, AT8-IR, or 4G8-IR were predictors of dependent variables WMLA or WML-BiA and LFB-IOD measures. All stepwise regression data had a VIF < 2, indicating no collinearity, and is presented in Table 3. Briefly, regarding WML severity, i.e., WMLA, SI was found to be a significant predictor of WMLA in both the control (*β = *0.644, *p = *0.002; Table 3a) and AD (; *β = *0.505, *p = *0.027; Table 3b) groups. Furthermore, in the AD group, a second model revealed frontal AT8-IR was also a significant predictor of WML when in the presence of SI (SI; *β = *0.515, *p = *0.014; AT8; *β = *0.437, *p = *0.032; Table 3b). Regarding demyelination, i.e., WML LFB-IOD, SI was found to be a significant predictor in both the control (*β = *0.766, *p = *0.0001; Table 3c) and AD (*β = *0.544, *p = *0.016; Table 3d) groups. No pathological variable was found to be a significant predictor of axonal density, i.e., WML-BiA (all models *p > *0.249).

**Table 3:** Stepwise linear regression data for independent predictors of white matter lesions severity and demyelination

a				
Controls				
Dependent variable = white matter lesion area				
Model 1 summary	*R*^2^	SE	*F*_(2)_	*p* value
	0.415	13.63	13.487	0.002
Independent variables	Standardized coefficients—*β*	*p* value		
SI	0.644	0.002		
CAA	–	0.994		
AT8-IR	–	0.945		
4G8-IR	–	0.995		
Age	–	1.000		

b				
AD				
Dependent variable = white matter lesion area				
Model 1 summary	*R*^2^	SE	*F*_(2)_	*p* value
	0.255	19.11	5.821	0.027
Independent variables	Standardized coefficients—*β*	*p* value		
SI	0.505	0.027		
CAA	–	0.981		
AT8-IR	–	0.999		
4G8-IR	–	0.997		
Age	–	0.892		
Model 2 summary	*R*^2^	SE	*F*_(2)_	*p* value
	0.446	16.98	6.444	0.009
Independent variables	Standardized coefficients—*β*	*p* value		
SI	0.515	0.014		
CAA	–	0.881		
AT8-IR	0.437	0.032		
4G8-IR	–	0.996		
Age	–	0.685		

c				
Controls				
Dependent variable = demyelination				
Model 1 summary	*R*^2^	SE	*F*_(2)_	*p* value
	0.587	37.77 E6	27.026	0.0001
Independent variables	Standardized coefficients—*β*	*p* value		
SI	0.766	0.0001		
CAA	–	0.994		
AT8-IR	–	0.944		
4G8-IR	–	0.995		
Age	–	1.000		

d				
AD				
Dependent variable = demyelination				
Model 1 summary	*R*^2^	SE	*F*_(2)_	*p* value
	0.295	53.50 E6	7.129	0.016
Independent variables	Standardized coefficients—*β*	*p* value		
SI	0.544	0.016		
CAA	–	0.981		
AT8-IR	–	0.999		
4G8-IR	–	0.997		
Age	–	0.892		

*SE*, standard error; *SI*, Sclerotic Index; *CAA*, cerebral amyloid angiopathy; *IR*, immunoreactivity; *AD*, Alzheimer’s disease

### Wallerian degeneration and ischaemia in WML

**Fig. 4:**
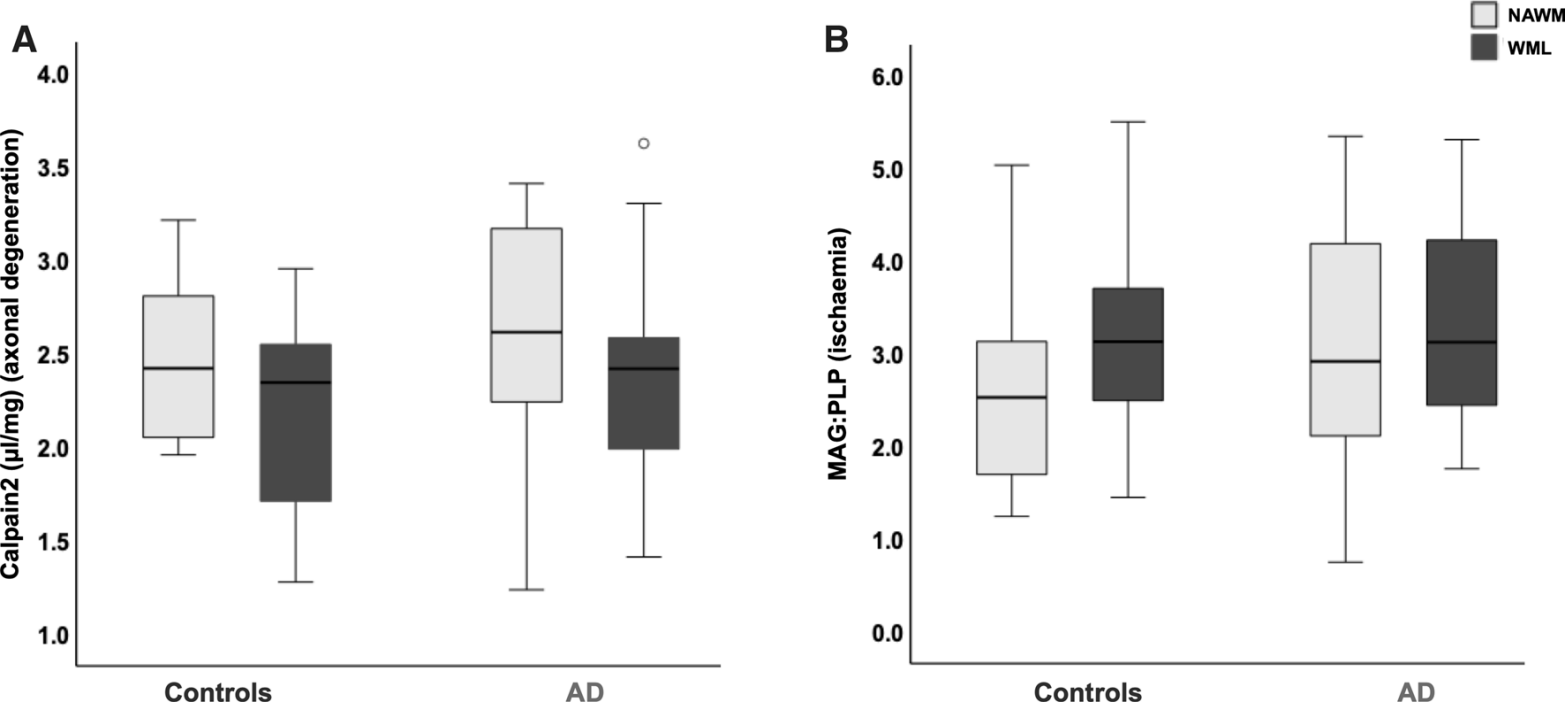
Box plots; **a** No significant differences were revealed in calpain2 measures between AD and controls nor between WML and NAWM measures within groups. No significant differences were revealed in MAG:PLP measures between AD and controls nor between WML and NAWM measures within groups. *p* values are shown in main text. *AD*, Alzheimer’s disease; *MAG*, myelin-associated glycoprotein; *PLP*, proteolipid protein

Regarding the Wallerian degeneration marker calpain2, no significant differences were seen in WML or NAWM measures between AD and controls (*p > *0.328; Fig. 4a). Furthermore, intragroup comparisons revealed no difference in calpain2 measures between NAWM and WML (both AD and controls *p > *0.503; Fig. 4a). Regarding the ischaemia marker MAG:PLP, no significant differences were seen in WML or NAWM measures between AD and controls (*p > *0.526; Fig. 4b). Furthermore, intragroup comparisons revealed no difference in MAG:PLP measures between NAWM and WML (both AD and controls *p > *0.087; Fig. 4b). Finally, we examined correlations of WML measures of calpain2 and MAG:PLP with SI, CAA, AT8-IR, and 4G8-IR. In the control group, no pathological variable was associated with WML-calpain2 (*p > *0.094) or WML-MAG:PLP (*p > *0.084; SI and WML-MAG:PLP, *p = *0.084). In the AD group, WML-calpain-2 was associated with 4G8-IR only (rho 0.484; *p = *0.047; other variables *p > *0.111), and no associations were found between MAG:PLP and any pathological variables (*p > *0.079). Stepwise linear regression, adjusted for age, was used to investigate if the independent variables SI, CAA, AT8-IR, or 4G8-IR were predictors of dependent variables of WML-calpain2 or WML-MAG:PLP; no model was revealed in the AD or control for any dependent variables (all models *p > *0.141).

## Discussion

In this pilot study, we reveal for the first time in human *post-mortem* tissue that the aetiology of frontal WML in AD is possibly the result of both SVD-associated mechanisms and degenerative changes secondary to the accumulation of AD pathology. This current data suggest a contrasting aetiology to WML in the parietal lobe of the same cohort that was primarily associated with degenerative changes, as described in our previous findings in McAleese et al. [30]. Combined with our previous findings, our current data reveal that regional differences in the aetiology of WML in AD with posterior WML possibly associated with degenerative mechanisms secondary to AD pathology, while anterior changes can be associated with both SVD and degenerative associated mechanisms.

It has been previously shown that parietal WML as seen in AD can occur as a consequence of degenerative axonal loss secondary to AD pathology in addition to SVD-associated white matter changes. Regional variation in the pathogenesis of white matter changes has been indicated using neuroimaging, but has not been investigated using human *post-mortem* tissue. We reveal that frontal WML as seen in individuals with AD are associated with SVD as indicated by arteriolosclerosis being associated with and a significant predictor of WML severity. This is in contrast to our previous findings in the parietal WM, where WML are associated with cortical neurodegenerative pathology but not SVD [30]. SVD was also associated with and a predictor of WML severity in the non-demented control group in agreement with the findings from the parietal region of the same cohort [30]. This striking difference between the anterior and posterior regions indicates that SVD perhaps has a more prominent influence on the development of frontal WML but not parietal WML in both AD and non-demented controls. WMH volume is associated with increased vascular risk [58] with the frontal white matter particular vulnerable to CVRF, i.e., hypercholesterolemia [43] and hypertension [18, 44] and vascular insults. This maybe due to the frontal WM blood supply being from the territories of the distal branches of the superficial perforating arteries of the anterior and middle cerebral arteries, which are prone to the development of SVD.

The underlying pathomechanisms of white matter changes in AD are still poorly understood and heterogeneous, comprising of a complex interface between neurodegeneration and vascular dysfunction. Although the causal effects are still elucidated, it is thought that cerebral hypoperfusion as a result of CVRF can lead to critical dysfunction of the neurovascular unit, BBB exchange, and clearance pathways, which can then leave the brain vulnerable to developing, or synergistically influencing, the development of concomitant neurodegenerative disease, ischaemia, and neuroinflammation [45]. Interestingly, we found no increase in the biochemical markers of ischaemia (MAG:PLP) in white matter lesion tissue compared to normal white matter, and only a trend was revealed between increasing arteriolosclerosis severity and ischaemia. This was surprising given that arteriolosclerosis, and in general SVD, is associated and a predictor of WML and is thought to be an ischaemia driven mechanism. This lack of difference in ischaemia may be due to the underpowering of the study, given that a previous study has found a strong association between MAG:PLP and arteriolosclerosis and SVD severity in two separate cohorts [5]. However, it is important to note that ischaemia in SVD can be a later event, especially when there is no concomitant CVD and/or disrupted cerebral blood flow. Earlier stage vascular dysfunction at the cellular level, i.e., BBB breakdown, perivascular leakage, endothelial dysfunction, and an impaired neurovascular unit, may result in white matter tissue damage before the onset of significant ischaemia, and therefore, these data might reflect an earlier stage of disease that is not yet primarily ischaemic (for a full review please see [55]).

However, this study also revealed evidence of a degenerative influence on the aetiology of frontal WML in AD. In the AD group, increasing severity of WML was associated with both axonal loss and trended with demyelination, in contrast to the non-demented control group in which WML were predominantly associated with demyelination only. This is reflective of the pathological composition of WML in the parietal region of the same cohort as shown in our previous study [30]. In addition, a correlation was revealed between calpain levels and Aβ pathology, possibly indicating an increase in calpain activation with progression of AD. The influence of AD-associated white matter changes has been previously indicated in neuroimaging data; increased Aβ pathology, as detected via PET or CSF, is associated with frontal WMH independent of tau PET [16, 54]. Furthermore, individuals with autosomal dominant AD, who are below the age of onset for major CVRF, indicate early global, inclusive of specific frontal, white matter changes that are associated with increased p-tau and decreased Aβ 1–42 levels in CSF samples [2]. A key finding of the degenerative influence in the frontal region of AD cases is that HPτ was only a significant predictor of WML severity when in the presence of SVD, therefore, indicating that the possible influence of HPτ-associated degenerative axonal loss is in addition to and secondary to the independent influence of SVD. This finding is in agreement with a previous neuroimaging study which indicated to regional differences in periventricular white matter hyperintensities between non-demented controls and AD due to an additive effect of vascular and neurodegenerative injuries [58].

One may speculate that these data are reflective of the ‘two-hit vascular hypothesis’ of AD that states a first initial insult or ‘hit’ from cerebrovascular damage, such as chronic hypoperfusion from SVD, is sufficient to initiate neuronal injury, which are subsequently more vulnerable to a second ‘hit’ from independent AD-pathology insults, e.g., accumulation of Aβ and HPτ pathology [39].

The topographical progression of HPτ pathology in AD reaches the frontal cortices in the later stages of the disease progression, requiring a longer time frame for a degenerative influence to become prominent, compared to the more localised parietal white matter that has direct white matter projections with the hippocampal and entorhinal subfields. Finally, lack of an increase in WML measures of calpain in the AD group indicates no significant upregulation of Wallerian degeneration, in contrast to the significant increase seen in AD WML tissue from the parietal region [30], further suggesting that significant degenerative insults occur after vascular insults. Therefore, SVD-associated mechanisms appear to exert a significant impact to the frontal white matter, compared to the parietal white matter, resulting in the regional aetiological differences. This highlights the importance of early intervention regarding CVRF and the consideration of vasculoprotective treatments.

Unexpectedly, no associations were seen between CAA and frontal WMH. White matter changes in both sporadic AD-associated CAA [11, 37] and familial APP mutations [57] have indicated an increase in frontal WMH burden, indicating that the co-existence of CAA in AD may increase the burden of WMH. Given the likely mixed aetiology of frontal WMH and that CAA represents an interface between cerebrovascular disease and neurodegenerative pathology, an association with CAA was speculated. Although CAA primarily affects the leptomeningeal and cortical vessels of the neocortical regions and somewhat resembles the distribution pattern of Aβ pathology, the occipital lobe is frequently and severely affected first before progressing rostrally [3]. A lack of relationship may reflect that CAA was not as severe in the frontal region of this cohort and the use of semi-quantitative assessment, in comparison to quantitative methods used for the other neuropathologies may not have been sufficient to reveal any associations with white matter changes. Use of advancing artificial intelligence systems for the quantification of CAA in future studies is warranted.

The pathological findings and suggestions from this current data and our previous study [30] regarding the regional specific pathogenesis of WML in AD are consistent with neuroimaging findings from Lee et al., which indicated regional aetiological differences in microstructural disruption of the CC [25]. Lee and colleagues indicate that both vascular and AD-associated degenerative mechanisms contributed to anterior CC white matter disruption which is in line with our data from this study of a mixed pathogenic model of frontal WMH development with the contribution of both SVD (primarily) and a later addition of AD-associated degenerative mechanisms. In contrast, Lee and colleagues demonstrate that posterior CC white matter disruption was primarily driven by AD-associated degeneration and not vascular mechanisms which is in agreement with our previous findings that parietal WML in AD are more likely associated with degenerative axonal changes secondary to cortical AD pathology and not SVD [30].

Understanding the various underlying pathogenic mechanisms of white matter changes and their regional differences is crucial for the accurate diagnosis and therapeutic interventions of patients with cognitive impairment. During life, white matter changes are detected as WMH and often interpreted as a surrogate marker for general SVD [13, 15], and, although the presence of white matter change is indicated, imaging changes are unable to determine the specific SVD-associated pathology or an alternative mechanism, i.e., degenerative axonal loss. Hence, elucidating and understanding the underlying pathology and associated mechanisms that underline identical WMH signatures are crucial. This study did not directly map WMH and with regions of pathology, and therefore, the proposed concept is speculative and warrants future confirmative direct radiological–neuropathological studies. It is crucial that location, severity, and progression gradient of WMH burden are taken into consideration when interpretating white matter changes, especially given the use of WMH as a surrogate biomarker for SVD which may result in the misdiagnosis of patients, bias cohort stratification in clinical trials, and have a detrimental impact on therapeutic interventions for patients.

One important limitation of this study is the modest dataset and possible under powering; due to the use of a consecutive series not all cases contained a WML, therefore, reducing the case numbers for statistical analysis. We employed pairwise deletion in the analysis to optimise the data available, but results should be interpreted as tentative, and a larger follow-up study for validation is encouraged. In additional, we are aware that the analysis has not taking into consideration important CVRF and CVD covariates i.e., blood pressure, presence of diabetes, hyperlipidaemia, hypercholesterolaemia, smoking, etc. We acknowledge that these factors, particularly hypertension, can influence the development and progression of both white matter changes and specifically arteriolosclerosis and lipohyalinosis [22, 24]; however, these data were unavailable or insufficient for analysis.

In conclusion, this preliminary study tentatively suggests that in individuals with AD, both SVD-associated mechanisms and cortical AD-associated degenerative pathology have influences on the development of frontal WML in contrast to parietal WML that appear to be more likely associated with AD-associated degenerative mechanisms.

## Supplementary Information

Below is the link to the electronic supplementary material.

Supplementary file1 (DOCX 32 kb)

## Abbreviations

AD: Alzheimer’s disease
Aβ: Amyloid-beta
BiA: Bielschowsky’s % area
CAA: Cerebral amyloid angiopathy
CC: Corpus callosum
CVD: Cardiovascular disease
CVRF: Cardiovascular risk factor
DTI: Diffusion tensor imaging
ELISA: Enzyme-linked immunosorbent sandwich assays
HPτ: Hyperphosphorylated tau
IOD: Integrated optical density
IR: Immunoreactivity
LFB: Luxol fast blue
MAG: Myelin-associated glycoprotein
NAWM: Normal appearing white matter
PLP: Proteolipid protein
RBG: Red, blue, green (threshold)
SI: Sclerotic Index
SVD: Small vessel disease
WMH: White matter hyperintensity
WML: White matter lesion
WMLA: White matter lesion % area
