# Supplementary material for: Frontal white matter lesions in Alzheimer’s disease are associated with both small vessel disease and AD-associated cortical pathology
Source: Acta Neuropathol. 2021 Oct 4;142(6):937–50. doi: 10.1007/s00401-021-02376-2 (PMC8568857; doi:10.1007/s00401-021-02376-2)
Supplement: Supplementary file 1 — Supplementary file1 (DOCX 32 kb) [file 401_2021_2376_MOESM1_ESM.docx]

**Supplementary table 1. Known cause of death and presence of CVRF or CVD within the cohort**

| **Case number** | **CNP diagnosis** | **Sex** | **Cause of death** | **Clinical assessment** | **MMSE (<18 month pre-death)** | **ApoE status** | **Presence of hypertension** | **Presence of diabetes** | **Smoking status** | **CVRF or CVD** |
| --- | --- | --- | --- | --- | --- | --- | --- | --- | --- | --- |
| 1 | AD | female | Pneumonia | Annual | 0 | E3,E4 | No | No | Non smoker | None |
| 2 | Control | female | Acute subdural haematoma | None - post hoc donor | - | E3,E3 | No | - | - | AF |
| 3 | Control | male | Myocardial infarction | None - post hoc donor | - | E3,E3 | - | - | - | Died of MI |
| 4 | Control | female | Lung cancer | None - post hoc donor | - | E3,E3 | No | - | - | IHD |
| 5 | Control | female | Left ventricle failure | Annual | 29 | E2,E3 | Yes | No | Non smoker | IHD |
| 6 | AD | female | Renal failure | None - post hoc donor | - | - | Yes | - | - | - |
| 7 | AD | male | Ischemic stroke | Occasional | 7 | E3/E4 | Yes | - | - | - |
| 8 | AD | male | Pneumonia | Occasional | 6 | - | - | - | - | - |
| 9 | Control | female | Aspiration Pneumonia | Annual | 30 | E3,E3 | No | - | - | IHD |
| 10 | Control | male | Not known | None - post hoc donor | - | - | - | - | - | - |
| 11 | AD | female | Aspiration pneumonia | None - post hoc donor | 0 | - | - | - | - | - |
| 12 | AD | female | Chronic obstructive pulmonary disease | None - post hoc donor | 22 | E3,E3 | - | - | - | - |
| 13 | Control | male | Pneumonia | Occasional | 26 | - | - | DMT2 |  | - |
| 14 | Control | female | Multi organ failure from ischaemic bowel | Annual | 29 | E3,E3 | Yes | DMT1 |  | Previous TIA |
| 15 | AD | male | Pneumonia | Annual | 1 | E4,E4 | No | No | Non smoker | - |
| 16 | AD | female | Pneumonia | Occasional | - | - | - | - | - | - |
| 17 | Control | female | Metastatic cancer | None - post hoc donor | - | E2,E3 | - | - | - | - |
| 18 | Control | female | Multi organ failure from ischaemic bowel | Annual | 30 | E3,E3 | No | - | - | IHD and AF |
| 19 | AD | female | Not known | None - post hoc donor | - | - | Yes | - | - | - |
| 20 | Control | female | Peritonitis | Annual | 25 | E3,E3 | Yes | - | - | - |
| 21 | AD | female | Pneumonia | Annual | 7 | E3,E4 | Yes | No | - | Angina |
| 22 | AD | male | Frailty of old age | Annual | 8 | E3,E4 | No | DMT2 | Former smoker | Statin |
| 23 | Control | male | Pneumonia with Infective endocarditis | None - post hoc donor | - | E2,E3 | No | - | - | Mitral valve replacement CABG |
| 24 | AD | male | Ischemic stroke | Annual | 0 | - | No | No | Former smoker | IHD Statin |
| 25 | AD | female | Pneumonia | Annual | 7 | E3,E3 | Yes | No | Non smoker | - |
| 26 | AD | female | Frailty of old age | Occasional | 18 | E3,E4 | - |  | Non smoker | - |
| 27 | AD | female | Pneumonia | Annual | 0 | - | Yes | No | Non smoker | - |
| 28 | AD | female | Not known | Occasional | 20 | E4,E4 | No | No |  | - |
| 29 | AD | male | Renal failure | Annual | 0 | E3,E4 | Yes | No | Former smoker | IHD MI Statin |
| 30 | AD | female | Pneumonia | Annual | 2 | E3,E4 | - | - | - | - |
| 31 | AD | female | Ischemic stroke | Occasional | 10 | E3,E4 | - | - | - | - |
| 32 | Control | male | Cerebral haemorrhage | Annual | 28 | E3,E4 | No | No | Non smoker | - |
| 33 | Control | female | Pneumonia | Annual | 22 | E3,E4 | Yes | No | Non smoker | - |
| 34 | Control | male | Renal failure | Annual | 20 | E3,E4 | Yes | No | Former smoker | - |
| 35 | Control | female | Heart failure | Occasional | 27 | - | No | No | Non smoker | Previous TIA |
| 36 | Control | male | Prostate cancer | Annual | 29 | E3,E3 | Yes | DMT2 | Former smoker | - |
| 37 | Control | male | Pulmonary embolism | Annual | 29 | E3,E3 | Yes | No | Former smoker | - |
| 38 | Control | male | Sepsis secondary to aspiration pneumonia | Annual | - | - | Yes | No | - | Previous TIA Statin |
| 39 | Control | male | Chronic lymphocytic leukaemia | Annual | 30 | E2,E3 | Yes | No | Non smoker | - |
| 40 | Control | female | Metastatic cancer | Annual | 30 | E3,E3 | Yes | DMT2 | Former smoker | Statin |

‘-‘ indicates data was not available or recorded. CVRF, cardiovascular risk factor; CVD, cardiovascular disease; CNP, cliniconeuropathological diagnosis; AD, Alzheimer’s disease; DMT1/2; diabetes mellitus type 1/2; AF, atrial fibrillation; MI, myocardial infarction; IHD, ischaemic heart disease; TIA, transient ischaemic attack
